# Three-year follow-up of a Traumatic Hemipelvectomy Survivor: A Case Report

**DOI:** 10.5704/MOJ.2111.024

**Published:** 2021-11

**Authors:** KL Wan, MS Azlan, AS Syed-Azmi, RTS Lattish, WI Faisham

**Affiliations:** 1 Department of Orthopaedics and Traumatology, Hospital Sungai Buloh, Sungai Buloh, Malaysia; 2 Department of Rehabilitation Medicine, SOCSO Tun Razak Rehabilitation Centre, Melaka, Malaysia; 3 Department of Orthopaedics, Universiti Sains Malaysia, Kubang Kerian, Malaysia

**Keywords:** traumatic hemipelvectomy, traumatic hemipelvis amputation, hemipelvectomy prosthesis

## Abstract

The management of a patient with traumatic hemipelvectomy is complex. We report the acute management and rehabilitation of a 21-year-old patient as well as her prosthesis modification. She was able to return to society as a K3 level ambulator.

## Introduction

A traumatic hemipelvectomy injury is commonly complicated with multiple injuries, insufficient soft tissue coverage, infection and wound healing issues. The largest series so far was 21 cases in which most survivors were young, and their outcomes were variable^1^.

We report the early management and a patient’s rehabilitation journey in achieving independence following this devastating injury.

## Case Report

A 21-year-old female motorcyclist collided with a four-wheel-drive vehicle. She was pinned under the wheel for 30 minutes before being extricated. On arrival at the emergency room, her Glasgow Coma Scale (GCS) was 15/15 and pupils were equal and reactive. Her heart rate was 110 beats/minute and blood pressure was 100/70mmHg. Her distal third right tibia was completely amputated. There was a large right inguinal wound extending into the perineal region, exposing the flail iliac crest. The right leg was insensate and popliteal pulse was absent. An immediate pelvic binder was applied. Focused Abdominal Sonogram in Trauma (FAST) was negative for intra-abdominal fluid. She was resuscitated with one litre 0.9% saline, five bags of pack cells (PC), and one cycle of disseminated intravascular coagulation (DIVC) regime. Initial haemoglobin and lactate levels were 7g/dL and 3.1mmol/L, respectively. Both renal and liver functions were within the normal range.

**Fig 1:**
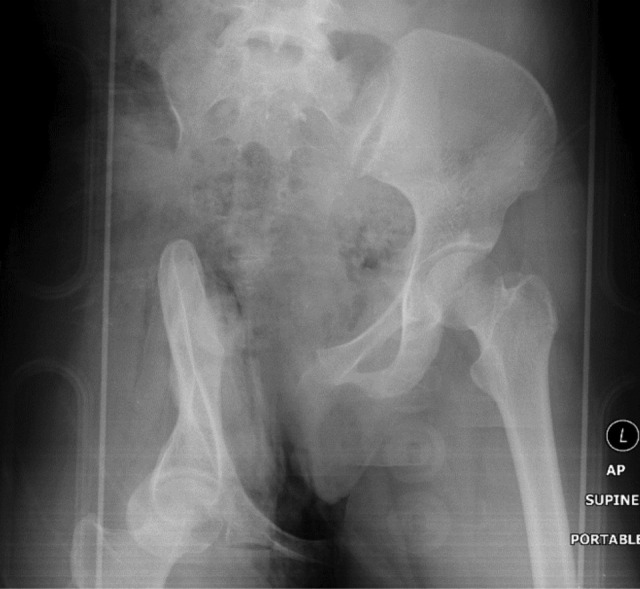
Pelvic anterior-posterior radiograph showed the survivor’s right hemipelvis was sheared inferiorly and malrotated.

**Fig 2:**
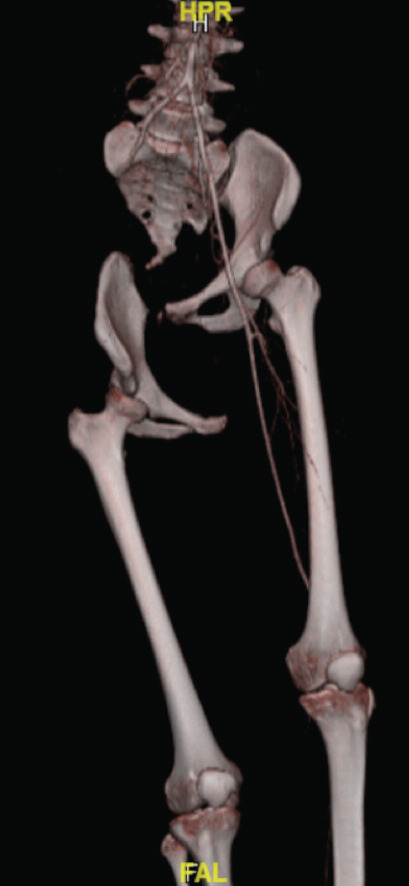
3D reconstructed Computer tomography angiogram (CTA) showed loss of arterial contrast flow at proximal right external iliac artery and right hemipelvis was sheared inferiorly.

The pelvic radiograph showed a wide distraction-separation of the right hemipelvis (Fig. 1). There was a loss of contrast flow after the right external iliac artery without contrast reconstitution distally on the computed tomography (CT) scan (Fig. 2). The ascending colon was contused and the right-sided pelvic floor muscles were disrupted with extension into the vagina wall and the rectal sphincter. CT brain showed right frontal subarachnoid haemorrhage and subdural haemorrhage with cerebral oedema. She also sustained an open comminuted fracture of the right olecranon, which was plated three weeks later.

Under general anaesthesia, surgical exploration was undertaken. Intra-operatively, both right external and internal iliac vessels were avulsed with stump thrombosis up to the right common iliac vessels. Both sciatic and femoral nerves were avulsed from the lumbosacral plexus. The vaginal wall and anal sphincter were lacerated and communicated with the perineal wound. The right hemipelvis was hanging by the skin as the entire right gluteus muscle was avulsed from its insertion.

The right common iliac vessels were ligated while superficial bleeding vessels on the bladder were secured with running sutures. The hemipelvis and its muscle attachment were detached from the posterior skin to create a random-pattern skin-fascia flap for soft tissue cover. The remaining pelvic floor muscles, rectum, and vaginal wall were repaired. A loop transverse colostomy on the left side of the abdomen was performed. She required another three bags of packed cells, fresh frozen plasma, and cryoprecipitate intra-operatively. Noradrenaline infusion was administered for 24-hrs to maintain haemodynamic stability.

Post-operatively, her wound healing was complicated with edge-flap necrosis and local infection, necessitating multiple attempts at wound debridement and subsequently, a local flap. The perineal wound developed into a rectovaginal-cutaneous fistula that required fistulectomy and debridement. A permanent colostomy was constructed due to a dysfunctional anal sphincter and failure of an attempt at reversal. The entire process of wound management took nine months before it fully healed.

The patient’s early rehabilitation was hampered by wound breakdown, nociceptive pain from the open wound, and a right olecranon fracture. Additionally, she developed worsening phantom limb pain after a month, when the wound had stabilised. She was bedbound for the first six weeks before she could tolerate incremental bed prop-up. She was then transferred to a recliner wheelchair with an anti-tipper and air cushion. Gradually, she managed to self-propel and transfer from bed-to-wheelchair independently. At three months, she started axillary crutches ambulation training in tripod gait. Prosthesis design was delayed until 18 months later due to wound complications.

**Fig 3:**
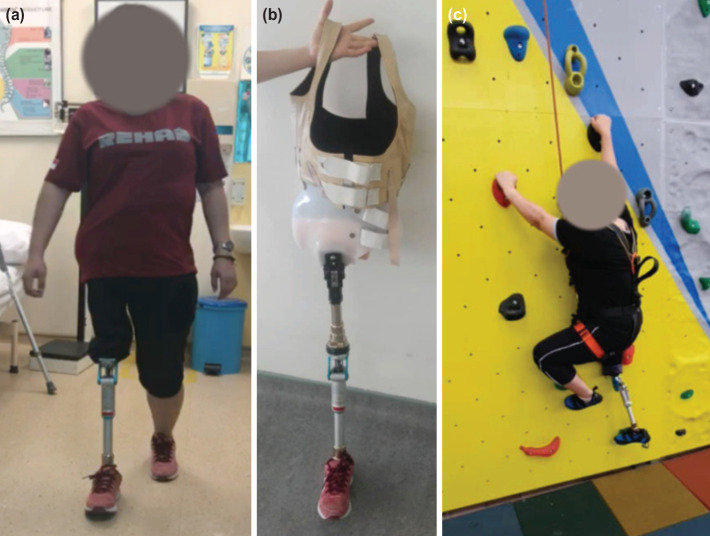
(a) The survivor ambulating with the prosthesis and (b) showing the prosthesis. (c) Patient was able to scale the rock wall with the prosthesis.

For prosthesis fitting, her right hemipelvis modular prosthesis included features such as trans-pelvic socket with single-axis hip joint, double-layer socket inlet lining with perlite and plastazote materials, body-corset-jacket type of suspension, and four-bar pneumatic swing control knee joint with a motion-control foot system. The body corset jacket suspension was modified to accommodate the colostomy. At three-year follow-up, she had joined the return-to-work programme and was tolerating short distance independent ambulation as a K3 level ambulator. K3 is defined as the ability to walk at a variable speed at the individual’s own pace and to traverse most environmental barriers beyond simple locomotion2. She wore the prosthesis for about twelve hours a day and even participated in a rock-wall climb (Fig. 3c).

## Discussion

In traumatic hemipelvectomy, haemorrhage control is the key to survival. Initial management options include pre-peritoneal packing, direct haemorrhaging vessel control, retrograde endovascular balloon occlusion of the aorta (REBOA), and angiographic embolisation^3^. Completion of traumatic hemipelvectomy allows good visualisation for haemorrhage control, evaluation of pelvic organs, and debridement for devitalised structures. The first published report of a 16-year-old male traumatic hemipelvectomy survivor in Malaysia was by Faisham *et al*^4^.

Following hemipelvectomy, the modalities for soft tissue reconstruction include a posterior flap with intact internal iliac and gluteal vessels or anterior flap by utilising external iliac and superficial femoral vessels for anteromedial myocutaneous flap^5^. The amputated leg is a donor option for a thigh musculo-cutaneous flap from various sites, for example, anteromedial thigh flap based on the superficial femoral artery (SFA) and fascio-cutaneous flap based on lateral circumflex femoral vessels that can be anastomosed to vascular stumps in hemipelvectomy^5^. However, in the emergency setting, good haemorrhage control and resuscitation remain the utmost priority over the soft tissue reconstruction decision. Faisham *et al* performed a gluteus maximus free flap by anastomosing avulsed inferior gluteal vessels to external iliac vessels in the same setting to achieve a viable gluteus muscle flap after the hemipelvectomy completion4. Avulsion type of vessel injury, extensive muscle damage, ischaemic time, and the availability of microsurgery facilities also need to be considered in all cases. In our case, the posterior skin flap based on random pattern vascularity was assessed to be viable and thus used as the cover. The flap survived with some amount of edge necrosis, infection, and fistulae complication.

The initial prosthesis fitting for our patient was difficult due to poor stability with waist level fitting, soft tissue complication, and colostomy. Initial phantom limb and neuropathic pain upon weight bearing further delayed the rehabilitation. Fig. 3a shows her ambulating with the prosthesis. Modifications made are highlighted in Fig. 3b. Table 1 shows the justifications for each modification. With that, the prosthesis provided comfort and stability for her to ambulate without support. She required 18 months of prosthesis fitting and training before successfully returning to society as a K3 ambulator^2^. Fig. 3c shows her performing rock-climbing activities.

**Table I:** Features of the patient’s right hemipelvis modular prosthesis and their justifications

Components	Specifications	Justifications
Socket	- Transpelvic socket and single axis hip joint - Front strap opening for easy donning and doffing and to secure the suspension system - Double layer reinforcements of the socket outer layer with polypropylene to prevent stump sliding - Socket inlet seating base padded with pelite layer and another layer of plastazote for added cushion, perineal region comfort and to minimise friction.	Transpelvic socket was used and modified for more push at the anterior and posterior walls. It concealed the stoma bag within the socket. A release pressure was modified at medial socket and groin regions which were resting on the anterior pubic bone and the posterior coccyx bone. Trim-line on the sound side was extended lower than the lateral margin to prevent or reduce lateral trunk bending during the gait trial. Single axis hip joint was chosen for the simplicity and stability during ambulation.
Suspension	Body corset jacket type: - Inner layer: lining sponge material - Middle layer: synthetic leather - Outer layer: jeans material	Body corset jacket type was able to reduce the pistoning effect and allowed extra suspension as this patient did not have a right iliac crest for socket suspension. A 7-point buckle system was made to ease the removal and change of the jacket for hygienic purpose. Third outer layer was stitched with jeans material for a firm hold of the inner lining sponge and the middle layer with synthetic leather.
Knee	4-bar with pneumatic swing control knee joint	Pneumatic knee joint was chosen because she was able to control the swing phase and cadence during walking. This system enabled the patient to tolerate fast or slow walking gaits with stable steps.
Foot	Motion control foot system	This system allowed plantar-flexion and dorsiflexion during walking so that she could adjust her stance.
Liner	Not applicable	
Cosmetics	Foam cover	For cosmetic appeal
Addition	Additional set of stocking for additional cosmetic appeal	

## References

[1] He Y, Qiu D, Zhou D, Li L, Wang B, Wang L (2019). Treatment of partial traumatic hemipelvectomy, a study of 21 cases.. J Bone Joint Surg Am..

[2] Balk EM, Gazula A, Markozannes G, Kimmel HJ, Saldanha IJ, Resnik LJ (2018). Lower Limb Prostheses: Measurement Instruments, Comparison of Component Effects by Subgroups, and Long-Term Outcomes. Rockville (MD): Agency for Healthcare Research and Quality (US). http://https://www.ncbi.nlm.nih.gov/books/NBK531523/.

[3] Tran TLN, Brasel KJ, Karmy-Jones R, Rowell S, Schreiber MA, Shatz DV (2016). Western Trauma Association critical decisions in trauma: management of pelvic fracture with hemodynamic instability-2016 updates.. J Trauma Acute Care Surg..

[4] Faisham WI, Azman WS, Muzaffar TMS, Muslim DAJ, Azhar AH, Yahya MM (2012). Traumatic hemipelvectomy with free gluteus maximus free fillet flap covers: A case report.. Malays Orthop J..

[5] Mat Saad AZ, Halim AS, Faisham WI, Azman WS, Zulmi W. (2012). Soft tissue reconstruction following hemipelvectomy: eight-year experience and literature review.. Science World Journal..

